# Supplementary material for: ProteinShader: illustrative rendering of macromolecules
Source: BMC Struct Biol. 2009 Mar 30;9:19. doi: 10.1186/1472-6807-9-19 (PMC2672931; doi:10.1186/1472-6807-9-19)
Supplement: Additional file 1 — ProteinShader program without source code. This compressed file contains the complete ProteinShader program including associated libraries, but no source code. A README.txt file gives an overview of the ProteinShader distribution, and the index.html file in the help subdirectory has directions on getting started with the program as well as a set of tutorials. [file 1472-6807-9-19-S1.zip › ProteinShader-beta-0_9_4-binary/help/api/org/proteinshader/structure/io/exceptions/class-use/PDBFieldExtractorException.html]

Uses of Class org.proteinshader.structure.io.exceptions.PDBFieldExtractorException (ProteinShader API)


|  |  |  |
| --- | --- | --- |
| **Overview** \| **Package** \| **Class** \| **Use** \| **Tree** \| **Deprecated** \| **Index** \| **Help** | |  |
| PREV   NEXT | **FRAMES**    **NO FRAMES**     **All Classes** |  |


---


## **Uses of Class org.proteinshader.structure.io.exceptions.PDBFieldExtractorException**

| Packages that use PDBFieldExtractorException | |
| --- | --- |
| **org.proteinshader.structure.io** | Holds the classes needed for reading Protein Data Bank structure files. |

Uses of PDBFieldExtractorException in org.proteinshader.structure.io

| Methods in org.proteinshader.structure.io that throw PDBFieldExtractorException | |
| --- | --- |
| `String` | `PDBAtomFieldExtractor.extractAltLocation(String line)`             Obtains the alternate location from (altLoc field: 17), or returns an empty String if it is not found (this field is usually blank). |
| `String` | `PDBAtomFieldExtractor.extractAtomID(String line)`             Obtains the atomID from (name field: 13-16). |
| `int` | `PDBAtomFieldExtractor.extractAtomSerialNo(String line)`             Obtains the atom serial number from (serial field: 7-11). |
| `AtomEnum` | `PDBAtomFieldExtractor.extractAtomType(String line)`             Obtains the atom type (chemical element) from an ATOM or HETATM record. |
| `String` | `PDBBetaStrandFieldExtractor.extractBetaStrandNumber(String line)`             Obtains the betaStrandNumber from (strandNo field: 8-10). |
| `String` | `PDBBetaStrandFieldExtractor.extractChainID(String line)`             Obtains the chainID from (initChainID field: 22). |
| `String` | `PDBHelixFieldExtractor.extractChainID(String line)`             Obtains the chainID from (initChainID field: 20). |
| `String` | `PDBAtomFieldExtractor.extractChainID(String line)`             Obtains the chainID from (chainID field: 22 ) if it is present. |
| `int` | `PDBAtomFieldExtractor.extractCharge(String line)`             Obtains the charge from (charge field: 79-80), or sets it to 0 if the field is only whitespace. |
| `List<Integer>` | `PDBConnectFieldExtractor.extractDstAtomSerialNo(String line)`             Obtains destination Atom serial number(s) from |
| `String` | `PDBBetaStrandFieldExtractor.extractEndResidueID(String line)`             Obtains the endResidueID by combining (endResName field: 29-31), (endSeqNum field: 34-37), and the optional (endICode field: 38). |
| `String` | `PDBHelixFieldExtractor.extractEndResidueID(String line)`             Obtains the endResidueID by combining (endResName field: 28-30), (endSeqNum field: 34-37), and the optional (endICode field: 38). |
| `String` | `PDBHelixFieldExtractor.extractHelixID(String line)`             Obtains the helixID from (helixID field: 12-14). |
| `double` | `PDBAtomFieldExtractor.extractOccupancy(String line)`             Obtains the occupancy from (occupancy field: 55-60). |
| `String` | `PDBAtomFieldExtractor.extractResidueID(String line)`             Obtains the residueID by combining (resName field: 18-20), (resSeq field: 23-26), and the optional (icode field: 27). |
| `String` | `PDBAtomFieldExtractor.extractResidueName(String line)`             Obtains the Residue name from (resName field: 18-20). |
| `int` | `PDBBetaStrandFieldExtractor.extractSense(String line)`             Obtains the sense (orientation) from (sense field: 39-40). |
| `String` | `PDBHelixFieldExtractor.extractSerialNo(String line)`             Obtains the helix serial number from (serial field: 8-10). |
| `String` | `PDBBetaStrandFieldExtractor.extractSheetID(String line)`             Obtains the sheetID from (sheetID field: 12-14). |
| `int` | `PDBConnectFieldExtractor.extractSrcAtomSerialNo(String line)`             Obtains the source Atom serial number from (serial field: 7-11) |
| `String` | `PDBBetaStrandFieldExtractor.extractStartResidueID(String line)`             Obtains the startResidueID by combining (initResName field: 18-20), (initSeqNum field: 23-26), and the optional (initICode field: 27). |
| `String` | `PDBHelixFieldExtractor.extractStartResidueID(String line)`             Obtains the startResidueID by combining (initResName field: 16-18), (initSeqNum field: 22-25), and the optional (initICode field: 26). |
| `int` | `PDBBetaStrandFieldExtractor.extractStrandsInSheet(String line)`             Obtains the number of strands in the sheet from (numStrands field: 15-16). |
| `double` | `PDBAtomFieldExtractor.extractTemperature(String line)`             Obtains the temperature from (tempFactor field: 61-66). |
| `HelixEnum` | `PDBHelixFieldExtractor.extractType(String line)`             Extracts the helix type from (helixClass field: 39-40). |
| `double` | `PDBAtomFieldExtractor.extractX(String line)`             Obtains the x-coordinate from (x field: 31-38 ). |
| `double` | `PDBAtomFieldExtractor.extractY(String line)`             extractY() - obtains the y-coordinate from (y field: 39-46 ). |
| `double` | `PDBAtomFieldExtractor.extractZ(String line)`             extractZ() - obtains the z-coordinate from (z field: 47-54 ). |

---


|  |  |  |
| --- | --- | --- |
| **Overview** \| **Package** \| **Class** \| **Use** \| **Tree** \| **Deprecated** \| **Index** \| **Help** | |  |
| PREV   NEXT | **FRAMES**    **NO FRAMES**     **All Classes** |  |


---

# *Copyright © 2007-2008*
